# Customized microscale approach for optimizing two-phase bio-oxidations of alkanes with high reproducibility

**DOI:** 10.1186/s12934-017-0788-4

**Published:** 2017-10-10

**Authors:** Johannes F. Kolmar, Oliver Thum, Frank Baganz

**Affiliations:** 1 Advanced Centre for Biochemical Engineering, Department of Biochemical Engineering, University College London, Bernard Katz Building, Gordon Street, London, WC1H 0AH UK; 2 Evonik Creavis GmbH, Paul-Baumann-Straße 1, 45772 Marl, Germany

**Keywords:** Scale-down, C–H activation, Whole-cell biocatalysis, Two-liquid phase, Monooxygenase, Reproducibility

## Abstract

**Background:**

Numerous challenges remain to achieve industrially competitive space–time yields for bio-oxidations. The ability to rapidly screen bioconversion reactions for characterization and optimization is of major importance in bioprocess development and biocatalyst selection; studies at conventional lab scale are time consuming and labor intensive with low experimental throughput. The direct ω-oxyfunctionalization of aliphatic alkanes in a regio- and chemoselective manner is efficiently catalyzed by monooxygenases such as the AlkBGT enzyme complex from *Pseudomonas putida* under mild conditions. However, the adoption of microscale tools for these highly volatile substrates has been hindered by excessive evaporation and material incompatibility.

**Results:**

This study developed and validated a robust high-throughput microwell platform for whole-cell two-liquid phase bio-oxidations of highly volatile n-alkanes. Using microwell plates machined from polytetrafluoroethylene and a sealing clamp, highly reproducible results were achieved with no significant variability such as edge effects determined. A design of experiment approach using a response surface methodology was adopted to systematically characterize the system and identify non-limiting conditions for a whole cell bioconversion of dodecane. Using resting *E. coli* cells to control cell concentration and reducing the fill volume it is possible to operate in non-limiting conditions with respect to oxygen and glucose whilst achieving relevant total product yields (combining 1-dodecanol, dodecanal and dodecanoic acid) of up to 1.5 mmol g_DCW_^−1^.

**Conclusions:**

Overall, the developed microwell plate greatly improves experimental throughput, accelerating the screening procedures specifically for biocatalytic processes in non-conventional media. Its simplicity, robustness and standardization ensure high reliability of results.

**Electronic supplementary material:**

The online version of this article (doi:10.1186/s12934-017-0788-4) contains supplementary material, which is available to authorized users.

## Background

**Fig. 1:**
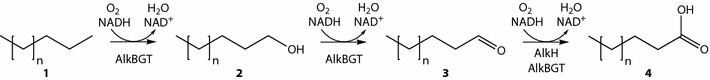
Reaction catalyzed by the AlkBGT monooxygenase; **1** *n*-alkane, **2** 1-alcohol, **3** aldehyde, **4** fatty acid; 1 ≤ n ≤ 12

The direct ω-oxyfunctionalization of aliphatic alkanes is efficiently catalyzed by monooxygenases, such as the di-iron, non-heme AlkBGT enzyme complex from *Pseudomonas putida*, under mild conditions (Fig. 1) [1]. This biocatalytic route offers exceptional selectivity with reduced energy demand, safety benefits and a reduction in synthesis steps compared to industrial chemical synthesis at elevated temperature and pressure [2]. The higher value products, primary alcohols, aldehydes and acids, are of considerable interest as intermediates in the chemical, pharmaceutical and cosmetics industries [3, 4].

Aqueous-organic two-liquid phase systems have long been employed in bioprocesses to supply organic substrates as well as extract inhibitory or toxic products back into the auxiliary organic phase [5–7]. However, despite recent advances, numerous challenges remain to achieve industrially competitive space–time yields and product titers for bio-oxidations [8]. To improve on this, the ability to rapidly screen bioconversion reactions for characterization and optimization is of major importance in biocatalyst selection and process development [9, 10]. Studies at conventional lab scale are time consuming and labor intensive with low experimental throughput. In recent years a range of sophisticated scale-down systems (< 10 ml) have emerged with new monitoring and control strategies to accelerate conventional bioprocess development [11]. The feasibility of parallel microwell systems to characterize and scale-up two-liquid phase whole-cell bioconversions has previously been shown for longer chain alkane substrates [12] and sitosterol [13]. However, excessive evaporation has limited the use of this microscale approach for highly volatile substrates.

Further, despite the significant negative economic impact associated with low reproducibility [14], there are only few studies that report the practicability and reproducibility of standardized scale-down microwell systems for complex applications in industrial biotechnology research. Either the extraction of compounds from commonly used laboratory plastics or the loss of volatiles can be a source of variability. Marques et al. [15] reported unidentified compounds in reactions from interactions of plate material with organic solvents; whereas Heinig et al. [16] reported the adsorption of analytes onto the plate material and extraction of plasticizers from polypropylene resulting in poor recoverability and reproducibility. Similarly, many of the optical online measuring techniques used in scale-down setups are incompatible with organic solvents diminishing the usefulness and applicability of these systems [17]. This is in addition to frequently reported location bias in microwell plates (MWPs) due to evaporation or temperature gradients in commercial MWP systems [18].

Thus, this study has a particular focus on investigating the impact of highly volatile substrates on conventional shaken microscale reactors and developed a microwell platform specifically customized for use with those. Particular attention was paid to material compatibility, evaporation and reliability, exemplarily using the whole-cell ω-oxyfunctionalization of aliphatic alkanes by AlkBGT in a two-liquid phase system.

## Methods

### Strains and plasmids

For all bioconversions *E. coli* GEC137 pGEc47ΔJ was used [19, 20]. pGEc47ΔJ contains all alkane oxidation genes of the OCT plasmid of *Pseudomonas putida* GPo1, except the alcohol dehydrogenase gene *alkJ*.

### Media composition

The aqueous growth medium used for fermentations was as described by Wubbolts, Favre-Bulle, and Witholt [21]: KH_2_PO_4_, 4 g l^−1^; K_2_HPO_4_ (3H_2_O), 15.9 g l^−1^; NH_4_Cl, 0.2 g l^−1^; (NH_4_)_2_SO_4_, 1.2 g l^−1^; Na_2_HPO_4_ (12H_2_O), 7 g l^−1^; l-proline, 0.6 g l^−1^; l-leucine, 0.6 g l^−1^; yeast extract, 5 g l^−1^ (all Sigma-Aldrich, UK); thiamine, 5 mg l^−1^ (Alfa-Aesar, UK); sterilised by autoclaving. The pH was adjusted to 7.2 before autoclaving. The following were heat sterilised and added subsequently: MgSO_4_ (7H_2_O), 1 g l^−1^; d-glucose, 10 g l^−1^ (all Sigma-Aldrich, UK); CaCl_2_ (2H_2_O), 0.04 g l^−1^; (Alfa-Aesar, UK); and 1 ml l^−1^ of the following trace minerals solution. Further, filter sterilised tetracycline, 15 mg l^−1^ (Sigma-Aldrich, UK) was added.

The trace minerals solution contained per litre of 5 mol l^−1^ HCl: FeSO_4_ (7 H_2_O), 40 g; MnSO_4_ (H_2_O), 10 g; CoCl_2_ (6H_2_O), 4.75 g; ZnSO_4_ (7H_2_O), 2 g; H_3_BO_3_, 0.5 g; MoO_4_Na_2_ (2H_2_O), 2 g (all Sigma-Aldrich, UK); CuCl_2_ (2H_2_O), 1 g (Riedel-de Haën).

For bioconversion reactions with resting cells a 150 mmol l^−1^ potassium phosphate buffer (pH 7.2) with thiamine (5 mg l^−1^) was supplemented with separately heat sterilized: MgSO^4^ (7H_2_O), 1 g l^−1^; d-glucose (all Sigma-Aldrich, UK); CaCl_2_ (2H_2_O), 0.04 g l^−1^ (Alfa Aesar, UK); and 1 ml l^−1^ of the trace minerals solution. Further, filter sterilized Triton X-100 (1 ml l^−1^) and tetracycline (all Sigma-Aldrich, UK) (10 mg l^−1^) were added.

### Bioconversion procedure in microwell plates

Bioconversions were carried out in either polytetrafluoroethylene (PTFE) (Radleys, UK) or polypropylene (PP) (Ritter GmbH, Germany) 24 deep square well (DSW) microwell plates (MWP). The plates were either sealed or a gas permeable sandwich cover (referred to as ‘permeable cover’ in the text) was used (Applikon, UK; Duetz et al. [22]).

For microwell bioconversions, buffered resting cells were used; bacteria were initially cultured in 2 l unbaffled shake-flasks. 200 ml fermentation medium were inoculated with 10 ml overnight culture. After 3 h (OD_600_ = 4–5) at 37 °C and 200 rpm, the temperature was dropped to 25 °C, followed by induction of AlkBGT expression with dicyclopropylketone (DCPK) (2.5 ml l^−1^) after another 30 min. After a further 18 h cells were aliquoted and harvested by centrifugation before resuspension in a potassium phosphate buffer. The bioconversion was then performed at 30 °C and 250 rpm with a cell concentration of 8.7 g_DCW_ l^−1^, 5.5 g l^−1^ glucose, a total fill volume of 350 μl and 20% alkane substrate, unless otherwise mentioned.

### Analytical procedures

#### Gas chromatography

Reactions in MWPs were first stopped by acidifying with 50 μl l^−1^ of 10 mol l^−1^ H_3_PO_4_, subsequently 100 μl ethyl acetate were added to the reaction mixture. After incubating on a thermomixer for 10 min at 50 °C and 1000 rpm the samples were centrifuged and the organic phase removed.

Organic samples were analyzed by GC-FID (Thermo Fisher Scientific, UK) equipped with a Rxi-5Sil MS column (30 m × 0.53 mm × 1.5 μm; Restek, USA) using helium as carrier gas (constant flow rate, 5 ml min^−1^). Octane bioconversions were analyzed with an injector and detector temperature of 250 and 280 °C respectively. The temperature program was set at 80 °C for 2 min, increased to 200 °C at 20 °C min^−1^ with a final hold of 3 min. Dodecane bioconversions were analyzed with an injector temperature of 270 °C. A temperature program of 100 °C for 1 min with an increase to 270 °C at 20 °C min^−1^ and a final hold of 1 min was used. Quantification was achieved using external standards. Reported concentrations are in relation to the aqueous reaction volume.

#### Liquid chromatography mass spectrometry

Alternatively, bioconversion product concentrations in MWPs were determined using a liquid chromatography mass spectrometry (LC–MS) method. Reactions were stopped by addition of 7 ml 80% (v/v) acetonitrile with 0.1% (v/v) formic acid. The liquid was transfer into a tube, vortexed and centrifuged. The supernatant was diluted in 80% (v/v) acetonitrile with 0.1% (v/v) formic acid, as required.

Liquid chromatography separation was carried out on an Agilent 1260 HPLC equipped with a Kinetex C18 column (100 mm × 2.1 mm column dimensions, 2.6 μm particle size, 100 Å pore size) and KrudKatcher Ultra HPLC pre-column filter (both Phenomenex, Aschaffenburg). The injection volume was 0.7 μl, the column temperature was set at 50 °C with a mobile phase flow rate at 0.6 ml min^−1^. Gradient elution was used (see Additional file 1: Additional data).

For mass spectrometry an Agilent 6410 triple quadrupole system was used with electrospray ionization in positive ion mode. Gas temperature was set at 280 °C, gas flow rate at 11 l min^−1^, nebulizer pressure at 50 psi and capillary voltage at 4000 V. Quantification of analytes was achieved with external standards spanning the range of sample concentrations, with the instrument either in selected-ion-monitoring (SIM) or multiple-reaction-monitoring (MRM) mode for detection. Reported concentrations are in relation to the aqueous reaction volume.

#### Oxygen measurements

Headspace oxygen levels in sealed MWPs were determined using a PSt1 needle mounted optical oxygen sensor connected to a TX3 light emitting diode and photodetector via a polymer optical fiber (all PreSens, Germany). Oxygen levels were recorded using the OxyView TX-6.02 software (PreSens, Germany). The sensor was calibrated at 0 and 100% air saturation in a stream of nitrogen and synthetic air respectively.

To mimic the oxygen consumption by microbial cells and bioconversion and test leak-tightness of sealed plates, the reaction of sodium sulfite to sodium sulfate catalyzed by Co(NO_3_)_2_ was used [23].

1.7 ml of a freshly prepared 30 g l^−1^ Na_2_SO_3_ solution were added to each well. 25 μl of a 1 g l^−1^ Co(NO_3_)_2_ catalyst solution in 0.5 mol l^−1^ HNO_3_ was transferred to the Na_2_SO_3_ solution in each well shortly before sealing the plate at the start of the experiment. Thus, the oxygen consumption capacity of the solution in each well was ≈ 20% more than present in the well headspace (see Additional file 1: Additional data). This was done to ensure completely oxygen free wells at the start of the experiment allowing for any additional dissolved oxygen and oxidation of Na_2_SO_3_ before the plate was sealed. The limited quenching capacity means that after the initial oxygen is removed from a well, any influx over the 48 h period would be measured at the end using the optical oxygen sensor.

#### Cell concentration

Dry cell weight (DCW) was determined in pre-weighed Eppendorf tubes by centrifuging two-phase samples of known volume at 13,000 rpm for 10 min. The supernatant was discarded and samples were dried at 100 °C for 24 h to 48 h until a constant weight was reached.

#### Sampling

Sampling from MWPs was routinely done using a sacrificial approach. Rather than taking small samples from one well over the duration of the experiment, complete well contents were sampled for each time point. Thus, when taking multiple samples at the same time point, these represent independent technical replicates. Where replicates were run, the number of samples (n) is reported together with the standard deviation (SD) in tables and figures.

### Design of experiments

Computer aided statistical design and analysis of experiments was carried out with Design-Expert 8 (Stat-Ease, USA). A response surface methodology (RSM) was used to gain detailed understanding of glucose, DCW and fill volume on 1-dodecanol and dodecanoic acid yields. Minimum and maximum levels for both factors were chosen based on initially collected data and previous understanding of the system. The number and value of the remaining levels were determined by an IV-Optimal algorithm to minimize the average variance of predicted responses throughout the design space. The factors were varied in a total of 48 experiments over seven levels each, including six replicate points.

Multiple linear regression was used to model the relationship between the factor and response data. Analysis of variance (ANOVA) was performed to validate the models in terms of fit and predictive power and test the significance of the model. All presented models are significant (p < 0.0001). Further diagnostics revealed no outliers in the data and response surface plots were subsequently used for model interpretation.

## Results and discussion

### Microwell setup for two-liquid phase biooxidations

#### Organic phase evaporation

The high vapor pressure of short-chain alkane substrates and products complicates their use with microscale tools. Specifically, it results in excessive evaporation when used in a microwell system with a permeable sandwich cover [12]. Despite the successful implementation of an optimized sandwich cover for the biooxidation of ethanol by Schlepütz and Büchs [24], the vapor pressure of alkane substrates is 2.5 × to 7.9 × higher in case of hexane and pentane (at 30 °C), resulting in higher and prohibitive levels of substrate evaporation. Consequently, a different measure that avoids escape of volatile substrates and products is required for use with high-throughput systems.

Commercially available designs aimed at parallel process development in the chemical industry use reflux condensing at small scale to control evaporation (Zinsser, Germany and HEL Group, UK). However, these solutions operate with round glass flasks and at their smallest volumes (≈ 10 ml), provide no external mixing. As previously found for shaken microwell plates, a square well geometry with pyramidal bottom provides satisfactory mixing and oxygen transfer due to the baffling effect of the perpendicular walls and the high surface area to volume ratio [25–27]. Thus, the relatively large volumes of the commercially available systems and their inferior mixing provision render them unsuitable for the heterogeneous mixtures of two-liquid phase whole-cell biocatalysis.

**Fig. 2:**
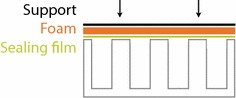
Schematic of microwell plate with sealing layers, pressure is applied uniformly as indicated by arrows

Instead, a simple seal was investigated that allows leak-tight closure of plates to prevent evaporation of organic substrates with high vapor pressure. This keeps the geometry of the previously developed and successfully scaled up system for longer chain alkane substrates whilst controlling evaporation [12]. The tested seal consists of three layers; an aluminum sealing film is mounted on a butyl rubber sheet backed by a sheet of silicone foam (Fig. 2). The layers were assembled into a stainless steel holder and pressed onto a MWP by fixing the holder and MWP between two aluminium plates.

**Fig. 3:**
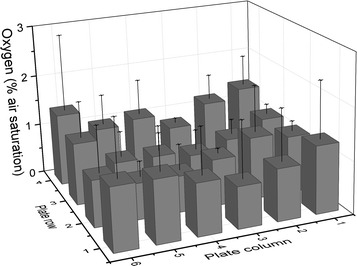
Oxygen ingress in individual wells of a plate sealed with clamp and filled with 1.7 ml Na_2_SO_3_ to provide oxygen free conditions initially. After 48 h at 37 °C, 250 rpm, endpoint measurements using a PreSens needle mounted sensor; n = 3, + SD

In order to test the tightness and uniformity of the seal, a needle mounted oxygen probe was used. This allowed the determination of oxygen levels in each well by piercing the seal with the needle and inserting the oxygen probe. To assess the seal, the influx of oxygen in initially oxygen free wells was measured after 48 h. Figure 3 shows that an adequately uniform distribution can be achieved over 48 h with oxygen ingress ranging from 0.6 to 1.4% into a sealed plate. In addition to containing oxygen, it is reasonable to assume that volatile alkane substrates are contained by the seal. Therefore, this approach can reproducibly seal a plate to the environment ensuring uniform conditions inside the plate.

#### Material compatibility

Especially at the microscale construction materials of tools for characterizing reactions in unconventional media are of great importance due to chemical compatibility challenges that are rarely reported in scale-down bioprocessing research. Moreover, due to the high surface-area-to-volume ratio of scale-down systems, any interactions are compounded. Initially, it was noted that standard thick-walled polypropylene (PP) plates were prone to discoloration after long-term use. Subsequent investigation showed that especially short-chain substrates such as pentane result in considerable softening and swelling of PP plates leading to deformation of the plate over the course of a 24 h bioconversion. With increasing chain length substrates, such as dodecane, have much reduced effects on PP.

Alternative materials of construction were considered in order to avoid any permeation or sorption interactions of substrate or products with the plate material. Here, virgin PTFE was investigated as a relatively easy to machine, reusable alternative that provides chemical inertness. 24 deep square well plates were machined from it. Other materials such as glass were not considered due to the difficulty of fabricating complex well geometries as well as its susceptibility to mechanical damage.

**Fig. 4:**
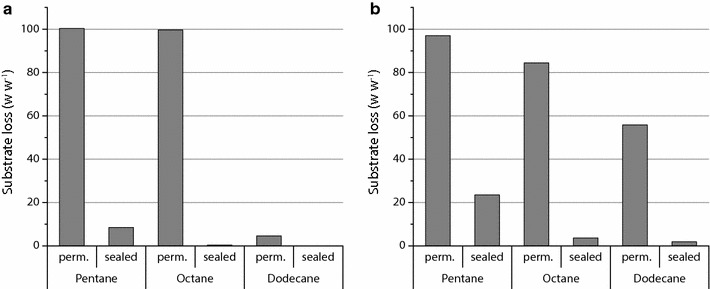
Substrate loss of n-alkanes from PTFE (**a**) or PP (**b**) plates with sealing or permeable covers filled with 500 μl alkane per well after 24 h at 37 °C and 250 rpm; Measured by percentage weight loss of alkane after incubation; Pentane filled in cold room at 4 °C

Figure 4 shows the loss by weight of alkane substrates from MWPs manufactured from PTFE or PP after 24 h. As can be seen from Fig. 4b, despite sealing a PP plate, slight substrate loss was measurable even for substrates with lower vapor pressure over 24 h, whereas in the PTFE plate no loss occurred with octane and dodecane (Fig. 4a). Only in case of pentane a loss of 8.5% occurred in the PTFE plate. Conversely, in a plate with permeable cover complete loss of n-alkanes with higher vapor pressure was recorded from PTFE plates, whereas 18% of octane was retained by weight in visibly dry PP plates. It is suggested that the reduced evaporation of octane from the PP plate with permeable cover is due to a proportion of the alkane substrate being absorbed by the polymer. This undesirable effect retards or prevents complete evaporation however it also removes the substrate from the reaction in the liquid phases. In case of dodecane, there was twelve times more loss from the PP plate with permeable cover than the PTFE plate, suggesting undesirable adsorption or permeation effects that could increase the effective surface area across which the alkane can evaporate resulting in higher evaporation rates than in the PTFE plate.

The superior properties of PTFE plates are due to its chemical inertness as well as its generally very low surface adhesion or adsorptive forces. The latter result in PTFE not only being hydrophobic but also lipophobic leading to a reduction in interactions with organic substrates. In contrast, PP is hydrophobic but lipophilic. Despite the hydrophobicity of PP and PTFE, Doig et al. [28] showed that surfactants in common aqueous fermentation media still enable wetting of surfaces of these materials to a similar extent. This is particular important for oxygen mass transfer [29].

To demonstrate the effects of the plate material, bioconversions in sealed plates made from PP and PTFE were compared. Table 1 compares the alcohol and acid product concentrations of hexane and octane bioconversions after 24 h. The results from the PP plates show consistently lower product concentrations compared to the PTFE plates with an average 2.2-fold reduction. Further, the aldehyde product concentrations are more affected with a 3.2-fold reduction in PP plates than the acid product concentrations with a 1.2-fold reduction. Although, it is unclear what effect is responsible for this specifically, a combination of the permeability or absorptive properties of the PP material as well as the change in reaction conditions due to the reduction in toxic organic concentrations is likely to be the cause.

**Table 1:** Alcohol and acid products from bioconversions in sealed PP and PTFE plates with hexane and octane substrates after 24 h

Substrate	Product	Plate material	Concentration (g l^−1^)
Hexane	1-Hexanol	PP	0.08 ± 0.003
		PTFE	0.16 ± 0.003
	Hexanoic acid	PP	0.26 ± 0.025
		PTFE	0.35 ± 0.000
Octane	1-Octanol	PP	0.11 ± 0.001
		PTFE	0.48 ± 0.006
	Octanoic acid	PP	0.26 ± 0.024
		PTFE	0.27 ± 0.000

n = 2, ± SD

The observed effects make PTFE a superior choice when using volatile organic substrates for bioconversions in MWPs. Compared to PP, using PTFE avoids interactions of organic substrates with the polymer MWP, thus avoiding an array of complications such as well-to-well carryover of organic substrates by permeation, reduction in substrate concentration by permeation or absorption, and leaching of compounds from the polymer.

#### Bioconversion in plates with sealing and permeable covers

**Fig. 5:**
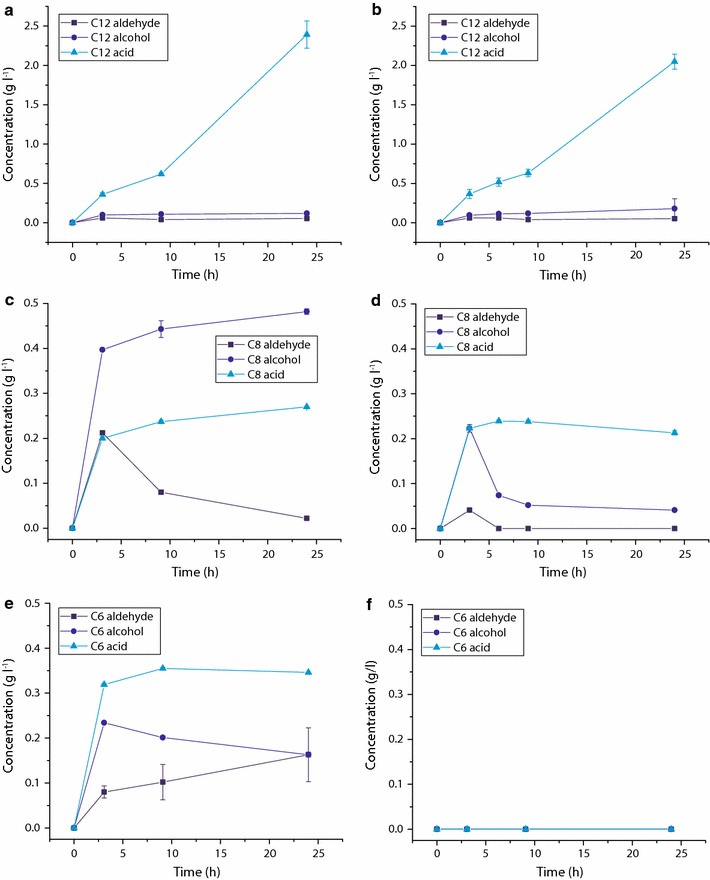
Bioconversion products of dodecane with sealed (**a**) and permeable cover (**b**), of octane with sealed (**c**) and permeable cover (**d**) and of hexane with sealed cover (**e**) and permeable cover (**f**) in PTFE plates over 24 h at 350 μl fill volume, 5.5 g l^−1^ glucose and 8.7 g_DCW_ l^−1^ cell concentration; n = 2, ± SD

To further illustrate the impact of the substrate evaporation on the reaction at microscale, 24 h time course bioconversions were run. Figure 5a, c show time course data in sealed PTFE plates for octane and dodecane, respectively. Furthermore, Fig. 5b, d allow the comparison to PTFE plates with a permeable sandwich cover. In case of octane, the comparison of Fig. 5c, d shows much reduced product concentrations in plates with permeable covers after 24 h despite initial similarity to plates with sealed covers. Especially the 1-octanol yield is 11.8-fold reduced in the plates with permeable cover after 24 h. Product loss by evaporation is the most likely reason for the lower concentrations of the more volatile products.

Not only are product concentrations directly influenced by evaporation, but also indirectly by changing process conditions due to reduced amounts of toxic products in the reaction. Thus the plates with permeable cover produce results for volatile short chain alkane products that are not representative of the actual reaction conditions and yields when evaporation is controlled. The results for the much less volatile dodecane substrate, on the other hand, show good similarity between the two different setups with the dodecanoic acid yield only 1.2-fold reduced in the plate with permeable cover (Fig. 5). Figure 5e shows time course data over 24 h for the hexane bioconversion in sealed plates. This substrate best illustrates the need for a seal since its high volatility, and of the respective products, results in no measurable bioconversion products when using a plate with permeable cover (Fig. 5f).

Thus, the comparison of plates with permeable cover to sealed plates shows that substrates with lower vapor pressure such as dodecane can be contained with a sandwich cover alone with good similarity between plates with permeable cover and sealed plates. However, in case of the more volatile octane and hexane, a seal is required to contain the volatile reaction substrates and products.

### Characterization of bioconversion in the sealed platform

A design of experiment (DoE) approach using response surface methodology (RSM) was adopted to systematically investigate the impact of sealing on the bioconversion and identify optimized, non-limiting conditions. For these experiments, dodecane was used as a model substrate due to its low volatility and good compatibility with analytical devices such as the oxygen sensor.

It was hypothesized that sealing plates may result in oxygen limitation for high-yield bioconversions. With no gas exchange to the outside in sealed plates, an oxygen consuming reaction inside a well has limited oxygen available. In order to identify limiting conditions three factors were varied in 48 runs with all samples taken after 24 h incubation at 30 °C and 250 rpm.

The first factor was cell concentration (DCW) (varied from 4 to 10 g_DCW_ l^−1^), which allows control over the intensity of the bioconversion hence avoid limiting conditions especially in terms of oxygen availability. Next, the control of glucose concentration (varied from 2 to 14 g l^−1^) allows optimization of co-factor regeneration and avoids catabolite repression depending on DCW. By reducing the total fill volume (varied from 250 to 600 µl), the ratio of available oxygen to culture volume increases thus avoiding limiting conditions. As the major product of the reaction, dodecanoic acid was used as the main response in these experiments. In addition, the remaining headspace oxygen concentration after 24 h was measured for each reaction.

**Fig. 6:**
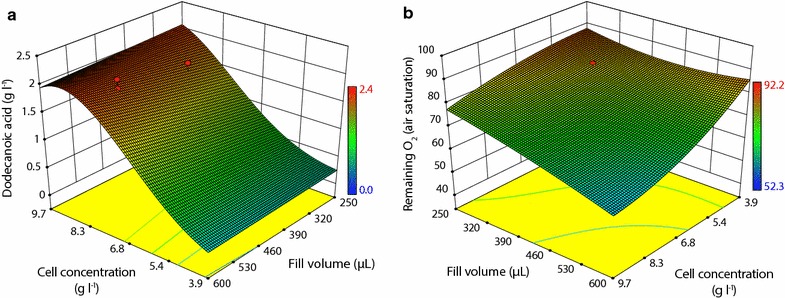
Influence of fill volume (μl) and cell concentration (g_DCW_ l^−1^) on dodecanoic acid yields (**a**) and oxygen remaining in a well (**b**), at 5 g l^−1^ glucose after 24 h at 30 °C, 250 rpm; graph taken from DesignExpert based on RSM data

Distinct optima in dodecanoic acid yields were found for the combination of factors. Generally, DCW has the largest impact on final dodecanoic acid concentration with highest yields at around 9 g_DCW_ l^−1^ (Fig. 6a). Fill volume has only moderate impact on yields in the range investigated here. With only a small influence, it is unlikely that changes in this parameter alter the flow regime and mixing in the shaken microwell. In fact, high-speed camera imaging revealed phase mixing and emulsion formation under the experimental conditions (see Additional file 1: Additional data).

When considering the residual oxygen concentration, Fig. 6b shows that with high DCW and high fill volumes oxygen levels reduce. Since no drop in yield was observed with high fill volume and high DCW (Fig. 6a), it is not expected that even at the reduced oxygen levels there is a limitation. However, it seems beneficial to reduce fill volumes to a workable level whilst keeping biomass concentrations in a certain range in order to avoid limiting conditions.

#### Operating window

**Fig. 7:**
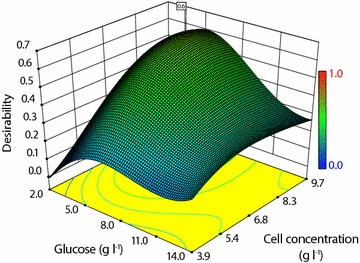
Desirability of conditions for glucose and cell concentration based on maximizing remaining oxygen and total product yield at 328 μl fill volume after 24 h at 30 °C, 250 rpm; graph taken from DesignExpert based on RSM data

**Fig. 8:**
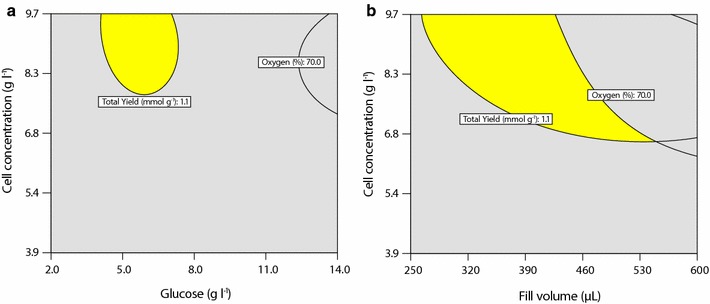
Operating window for cell concentration (**a**) and fill volume (**b**) at 328 μl fill volume and 5.4 g l^−1^ glucose respectively, regions marked in yellow achieve minimum 1.1 mmol g_DCW_^−1^ specific product yield and 70% remaining oxygen; graph taken from DesignExpert based on RSM data

Based on the RSM study, experimental conditions were optimized towards increasing the total product yield and increasing the remaining oxygen concentration in each well. A clear optimum can be seen in Fig. 7 at 5.4 g l^−1^ initial glucose, 8.7 g_DCW_ l^−1^ DCW and 328 μl fill volume resulting in 1.2 mmol g_DCW_^−1^ total product yield (combining 1-dodecanol, dodecanal and dodecanoic acid) and 76% remaining oxygen. Based on this information, a window of operation was defined with a minimum 1.1 mmol g_DCW_^−1^ total product yield and 70% remaining oxygen for the three factors. The highlighted areas in Fig. 8 show the combinations of DCW with glucose (Fig. 8a) and DCW with fill volume (Fig. 8b) that exceed the defined minimum. Thus, conditions for operation of the microwell platform were defined by reducing total fill volume to 350 μl, maintaining glucose at 5.5 g l^−1^ and DCW at 8.7 g l^−1^. Under these conditions, the bioconversion yield has a stoichiometric oxygen demand of 13.0 μmol, 14.0% of the theoretically available oxygen in the headspace (see Additional file 1: Additional data for calculation). Further, biocatalyst activities of 0.01 g_1-dodecanol_ g_DCW_^−1^ and 0.22 g_dodecanoic acid_ g_DCW_^−1^ can be achieved under these conditions. The values compare well with results previously obtained in stirred tanks with growing cells [12]. Thus, these conditions provide significant product levels, whilst ensuring non-limiting conditions in terms of oxygen and optimum amounts of glucose.

However, there is a chance of limitations when using this platform for further strain improvement work such as enzyme engineering, that often sees major yield improvements and consequently higher consumption of oxygen and carbon sources. These limitations can be avoided by reducing the deployed cell concentration or by further measures such as increasing the oxygen available by using oxygen enriched air or oxygen carriers such as perfluorodecalin [30, 31].

### Assessment of system reliability

In order to assess the robustness of the developed microwell system, replicate reactions under the non-limiting conditions were run, grouped and compared to each other using the one-way ANOVA method. For this analysis raw gas chromatograph peak area values (pA min) of alcohol and acid products for each well were used as exemplary results for a typical run.

It needs to be considered that the ANOVA analysis not only captures the variability due to the characteristics of the microwell setup, but also the inherent variability of the biological reaction and the variability introduced during sample handling and subsequent GC analysis. All data points were used and no modifications were made to the data. All group data was verified to be normally distributed and of equal variance.

Two comparisons were made (Table 2): the product concentrations of two sets of 24 replicate octane and dodecane biooxidations in two plates were compared. Second, the replicates from the 16 edge wells facing outside to the eight inside wells of a single plate were compared. Further, the coefficient of variation (CoV) of 24 replicate reactions in a single plate was determined. The CoV shows very low variation of results in case of the dodecane bioconversion (5.2% with dodecanoic acid), in reactions with octane the variation is higher likely due to the more volatile reactions compounds and lower concentrations. However, the deviations between replicates are still not significant (α = 0.01). Overall, the entire process and methodology shows good reproducibility. Comparison values in literature are scarce. However, Linde et al. report CoVs for metabolite production by filamentous fungi in 24-well plates using offline HPLC analysis [32]. The authors determined comparable CoV values after 48 h from six replicates. For ethanol, a product with high vapour pressure, the authors found a CoV of 22%. In case of products with low vapour pressure CoVs of 13 and 5% were found for citric acid and glycerol, respectively. Overall, the microwell plate system presented in this study compares well to these values.

**Table 2:** Statistical parameters from one-way ANOVA comparing bioconversion product concentrations of replicate biooxidations of octane or dodecane in either two separate PTFE plates or outside with inside wells of a single PTFE plate; coefficient of variation (CoV) of 24 replicate reactions in single plate; after 24 h at 328 μl fill volume, 8.7 g_DCW_ l^−1^ cell concentration and 5.4 g l^−1^ glucose, 30 °C, 250 rpm

Comparison	Two separate plates		Inside to outside wells of single plate		CoV (%)
Variable	*F*-statistic	*p* value	*F*-statistic	*p* value	
1-Octanol	2.15	0.15	0.60	0.45	16.8
Octanoic acid	4.09	0.05	0.59	0.45	17.2
1-Dodecanol	6.14	0.02	0.48	0.50	9.7
Dodecanoic acid	0.56	0.46	0.11	0.74	5.2

In order to further test the reliability and robustness of the developed system, the microwell system was transferred to an industrial laboratory. The focus was on ensuring easy transferability of the system without compromising reproducibility due to changes in operational variables such as the experimenter, analytical methods and consumables. To this end, replicate bioconversions of dodecane were run. A liquid chromatography mass spectrometry method was used for reaction product detection and quantification.

Table 3 shows the coefficient of variation for replicate dodecane bioconversions. The collected data shows very low variability between wells with coefficient of variation below 5%, supporting the reproducibility claims of the system. Thus, this transfer provides further evidence of the robustness and good reliability of the developed microwell system.

**Table 3:** Coefficient of variation (CoV) for dodecane bioconversion products during system transfer, after 6 h and 25 h at 10.5 g_DCW_ l^−1^ cell concentration and 15 g l^−1^ glucose, 30 °C, 250 rpm

Product	CoV (%) at	
	t = 6 h	t = 25 h
1-Dodecanol	nd	2.40^a^
Dodecanoic acid	1.95	3.16

n = 6, ± SD

nd, not determined

^a^ n = 2

## Conclusions

Although MWPs have been successfully applied to two-liquid phase systems before, the use of highly volatile short-chain alkanes was shown to be problematic with current scale-down tools. To improve material compatibility with the organic phase, MWPs machined from PTFE were investigated for these bioconversions. In contrast to PP, PTFE does not show any interaction with the liquid phases allowing reliable time resolved monitoring of bioconversion products. This reduces the likelihood of systematic errors occurring undetected. In combination with a sealing clamp, no significant variability such as edge effects could be determined (α = 0.01) whilst containing even highly volatile substrates and products.

A systematic statistical approach was adopted to characterize the system and show that a significant space of the experimental conditions results in non-limiting conditions with over 70% oxygen left after a 24 h bioconversion in sealed plates. By reducing fill volume and keeping DCW in a certain range, it is possible to operate in optimized, non-limiting conditions with respect to glucose and oxygen supply.

The developed MWP protocol greatly improves experimental throughput, accelerating the screening procedures specifically for two-liquid phase biocatalytic processes. It allows the rapid investigation of numerous parameters such as host strains, biocatalysts, substrates and reaction media in early process development and catalyst selection. The accelerated data collection on biological and process options allows obtaining key process design data early on, de-risking and speeding up the translation of new processes from laboratory to pilot-plant scale. Its simplicity and robustness result in a user-friendly system making it highly relevant for routinely characterizing bioprocesses in non-conventional media.

## Additional file

**Additional file 1.** Additional data.
