# Supplementary material for: Customized microscale approach for optimizing two-phase bio-oxidations of alkanes with high reproducibility
Source: Microb Cell Fact. 2017 Oct 10;16:174. doi: 10.1186/s12934-017-0788-4 (PMC5634833; doi:10.1186/s12934-017-0788-4)
Supplement: Supplementary file 1 — Additional file 1. Additional data. [file 12934_2017_788_MOESM1_ESM.docx]

**Customized microscale approach for optimizing two-phase bio-oxidations of alkanes with high reproducibility**

Johannes F Kolmar, Oliver Thum, and Frank Baganz

**Additional data**

**Liquid chromatography mass spectrometry**

The mobile phase for liquid chromatography consisted of Eluent A (1 ml l^−1^ aqueous formic acid) and Eluent B (0.80 ml ml^−1^ acetonitrile with 1 ml l^−1^ formic acid). Gradient elution according to Table 1 was used.

Table 1: Gradient elution for LC-MS method

| **Time** | **Eluent A** | **Eluent B** |
| --- | --- | --- |
| min | % | % |
| 0.0 | 77 | 23 |
| 0.3 | 77 | 23 |
| 0.4 | 40 | 60 |
| 2.5 | 40 | 60 |
| 2.6 | 2 | 98 |
| 5.5 | 2 | 98 |
| 5.6 | 77 | 23 |
| 9.0 | 77 | 23 |

**Oxygen measurements and calculation of theoretical oxygen consumption**

The amount of oxygen gas in a well can be estimated using the equation:

$$n_{O_{2}}=V_{O_{2}}\times\rho_{O_{2}}\times M_{O_{2}}$$

When filled with a 350 µl reaction volume a well with typically 11 ml total volume contains about 93 µmol molecular oxygen (filled at 20 °C and 0.101 MPa).

The amount of oxygen consumed by the oxidation of sodium sulfite to sodium sulfate can by calculated from the stoichiometric equation:

$$2{Na}_{2}{SO}_{3}+O_{2}\underset{\to}{Co\left( NO_{3} \right)_{2}}2{Na}_{2}{SO}_{4}$$

Assuming yields on oxygen for the alcohol, aldehyde and acid product (Y_P/O2_) of 1.0 mol mol^−1^, 0.5 mol mol^−1^ and 0.3 mol mol^−1^, respectively, the oxygen consumption of a reaction can be calculated by summing the consumption for each product of the reaction. Comparison to the oxygen available in a well gives the relative consumption:

$$\sum\left( n_{P}\div Y_{P/O_{2}} \right)$$

**Phase behavior during mixing**

In order to identify phase behavior and mixing of two-liquid phase reaction mixtures, cell-free two-liquid phase media were shaken with a 25 mm throw in transparent acrylic microwell mimics (1 cm side length) and recorded using a high-speed camera (Photron FASTCAM MC2 camera with a macro lens AF-S Zoom-Nikkor 28-70mm f/2.8D IF-ED).

Figure 2 shows the agitated two-liquid phase media for bio-oxidation of octane and dodecane. The figure shows droplet formation in shaken alkane/aqueous buffer mixtures upon addition of the surfactant Triton X- 100, similar to results from Grant et al. (2012). Overall, the results confirm previous findings that Triton X-100 addition promotes droplet formation and with that emulsion formation can be achieved in shaken square microwells.


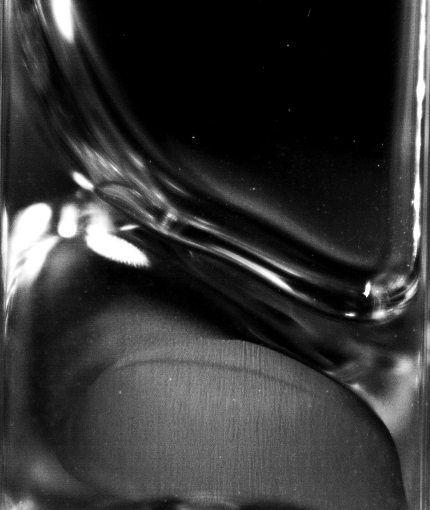

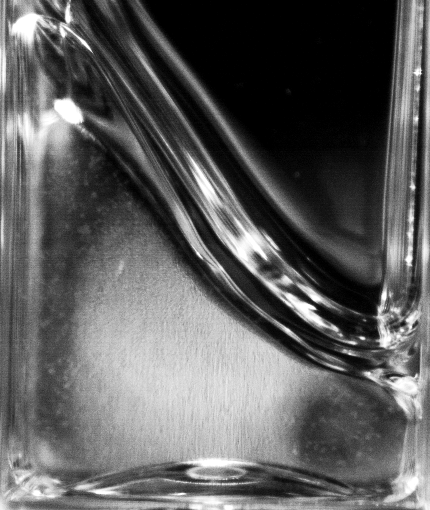


**A**

**B**


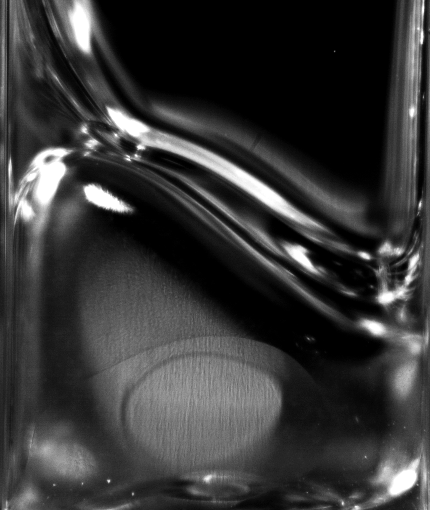

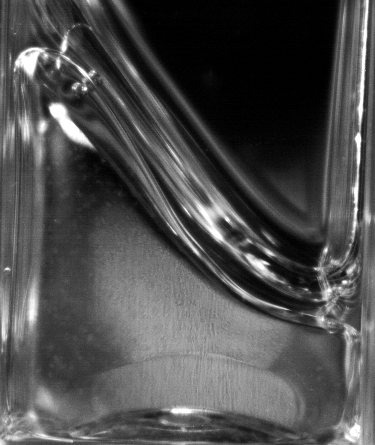


**C**

**D**

Figure 1: Mixing visualization of two-phase bioconversion media in well mimic with octane at 250 rpm (A), with octane with 0.1 % Triton X-100 at 250 rpm (B), with dodecane at 250 rpm (C), with dodecane with 0.1 % Triton X-100 at 250 rpm (D); all at 30 °C with 250 µl aqueous reaction buffer and 70 µl organic phase.
